# Estimation of Tegaserod Maleate by Differential Pulse Polarography

**DOI:** 10.4103/0250-474X.51956

**Published:** 2009

**Authors:** S. J. Rajput, H. A. Raj

**Affiliations:** Quality Assurance Laboratory, Pharmacy Department, Faculty of Technology and Engineering, The M. S. University of Baroda, Vadodara-390 001, India

**Keywords:** Differential pulse polarography, hanging mercury drop electrode

## Abstract

A highly sensitive differential pulse polarographic method has been developed for the estimation of tegaserod maleate after treating it with hydrogen peroxide solution. The oxidation of tegaserod maleate is a reversible process as the oxidized product could be reduced at hanging mercury drop electrode in a quantitative manner using differential pulse polarography mode. The limit of quantification was 0.1ng/ml. The voltametric peak was obtained at -1.05 volts in presence of 0.1M potassium chloride as supporting electrolyte. The technique could be used successfully to analyze tegaserod maleate in its tablet formulation.

Tegaserod maleate (TM), 3,5-methoxy-1H-indole-3-yl-methylene)-N-pentylcarbazimidamide hydrogen maleate[1], is a selective 5-HT4 receptor partial agonist with promotile activity in the gastrointestinal tract. The drug is not official in any Pharmacopoeia[2–4]. A thorough literature survey revealed a few HPLC-MS[5,6], HPLC[7] and spectrophotometric[8,9] methods for the analysis of TM in plasma and formulations. Voltametry offers a group of highly selective and sensitive methods. Some reports are now available in literature[10,11] using these techniques for analyzing the compounds of pharmaceutical interest. In the present study, TM was analyzed in bulk and in tablet formulation by differential pulse polarography after treating it with hydrogen peroxide.

A Metrohm 757 Computrace VA voltameter linked to a computer was used for voltametric measurements. The multimode electrode Metrohm stand was used in hanging mercury drop and dropping mercury drop electrode mode. The three electrode system was completed by means of a saturated calomel electrode as reference electrode and a platinum wire auxillary electrode. The aqueous 0.1 M potassium chloride was prepared as per IP procedure. All the aqueous solutions were prepared in double distilled water.

Standard tegaserod maleate was obtained as a gift sample from Torrent Research Centre, Ahmedabad and was used as such without any purification. The commercial tablet formulation Tegib-6 (Torrent Pharmaceuticals, Ahmedabad, India) was procured from local pharmacy.

Ten milligrams of standard TM was refluxed with 5 ml 3% H_2_O_2_ for 30 min at 80°. The flask was brought to room temperature then boiled again for 15 min without using the reflux condenser to expel the excess of H_2_O_2_. The solution was cooled and 10 ml of methanol was added. The solution was boiled again for 3-4 min to remove the traces of hydrogen peroxide. The solution was cooled again and diluted to 100 ml with methanol. The solution was diluted further to get a stock solution of 1 μg/ml. Standard solutions of other concentrations were prepared in similar manner.

Twenty millilitres of 0.1 M KCl was taken in the polarographic cell and nitrogen was purged through the solution for 300 s. An aliquot (1 μl-1 ml) from the standard TM solution was then added to KCl and purging of nitrogen was continued for 10 s. The differential pulse mode was used with pulse amplitude of 50 mV and a drop time of 0.8s. The polarogram was recorded after a sweep equilibration time of 10 s.

Twenty tablets (Tegib-6 of Torrent Pharmaceuticals) were triturated to a fine powder and weighed. Powder equivalent to 10 mg was weighed and treated with 5 ml of 3% H_2_O_2_ as described above. The estimation was done using standard addition method by adding the standard TM solution at two levels.

It was observed that TM is easily oxidized so we attempted to develop a voltametric method using glassy carbon electrode, having attempts to get a voltammogram using glassy carbon electrode or the anodic range of DME were unsuccessful. But the TM solution already treated with hydrogen peroxide gave a sharp polarogram in the cathodic range of dropping mercury electrode. Therefore the polarographic method was developed in the differential pulse polarographic mode using hanging mercury drop electrode (HMDE). The peak was obtained at -1.05 V (fig. 1). The effect of the composition of supporting electrolyte on the shape of polarogram of TM was studied and different media tried were Britton-Robinson buffers (pH 6-12), phosphate buffer (pH range 2-7) and 0.1 M lithium chloride but 0.1 M KCl gave the best results in the form of a sharp and well defined polarogram. Using 0.1M KCl as supporting electrolyte, working parameters of differential pulse polarography were established and effect of deposition potential, pulse amplitude and voltage step time were studied. The best polarogram was obtained with deposition potential -0.5 V, deposition time 68 s, drop size 4, equilibration time 10 s, voltage step 0.006 V, pulse amplitude 0.05 s and voltage step time 0.4 s. These optimized parameters are shown in Table 1. As the polarogram was smooth, Triton-X 100 or any other surfactant was not evaluated.

**Fig. 1:**
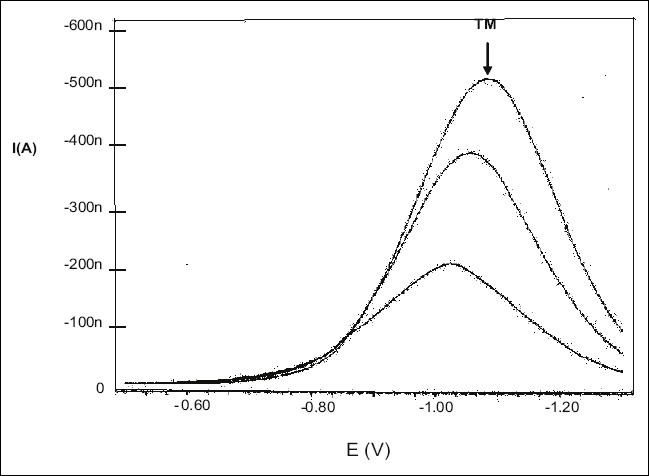
Differential pulse polarogram of tegaserod maleate The three polarograms are for three different concentrations (0.2, 0.4 and 0.6 μg/ml) of tegaserod maleate solution.

**TABLE 1:** WORKING CONDITIONS FOR DIFFERENTIAL PULSE POLAROGRAPHIC ANALYSIS OF TEGASEROD MALEATE

Parameter	Value
Electrode	HMDE
Deposition Potential	−0.5 V
Deposition time	68 s
Drop size	4
Equilibration time	10
Voltage step	0.0060 V
Pulse amplitude	0.05 V
Pulse time	0.05 s
Voltage step time	0.4 s

The polarogram was obtained in presence of 0.1 M KCl supporting electrolyte

The reproducibility of polarographic response was evaluated and relative standard deviation was calculated to be 0.56% for six determinations. The limit of quantification was 0.1 ng/ml, estimated as 0.1 μg/μl. The technique was applied to estimate TM in pharmaceutical preparations by standard addition method (fig. 2). The results showed 100.32±0.68% recovery suggesting results to be in good agreement with the label claim. The method though an indirect method but is highly sensitive and can be used to monitor the very small concentrations of TM.

**Fig. 2:**
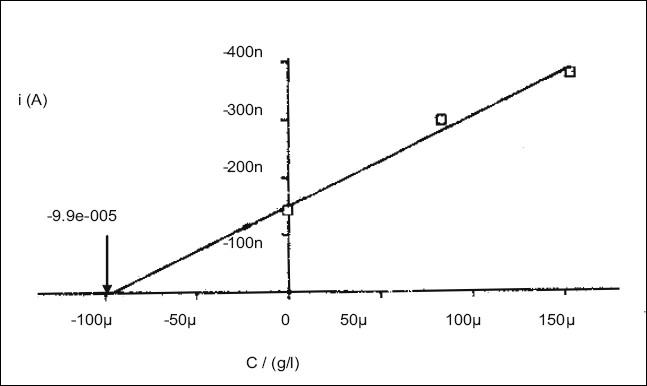
Analysis of tegaserod maleate by standard addition method The standard addition was done at two levels.

## References

[1] Budavari S (2001). The Merck Index.

[2] (1996). Indian Pharmacopoeia.

[3] (2002). British Pharmacopoeia.

[4] (2001). United States Pharmacopoeia.

[5] Zhou H, Khaliliesh S, Campestrine J, Appel-dingemanse S, Lachman L, Mcleod J (2000). Effect of gastric pH on plasma concentration of tegaserod. J Gastroenterol.

[6] Singh SS, Patel HV, Sharma K (2006). Estimation of tegaserod in human plasma by HPLC-Tandem mass spectroscopy and its application to bioequivalence study. Anal Chim Acta.

[7] Yang B, Gao MJ, Duan GL (2006). Ion Pair RPLC of tegaserod maleate and its impurities in pharmaceutical formulation and in dissolution studies. Chromatographia.

[8] Rajput SJ, Raj HA (2007). Assay of tegaserod maleate by difference spectroscopy. Indian J Pharm Sci.

[9] Rajput SJ, Raj HA (2007). Spectrophotometric estimation of tegaserod maleate in bulk drug and in tablet formulation. Indian J Pharm Sci.

[10] Inam R, Mercan H, Yilmaz E, Uslu B (2007). Differential pulse polarographic determination of moxifloxacin hydrochloride in pharmaceutical and biological fluids. Anal Lett.

[11] Debnath C, Haslinger E, Likussar W, Micheliletsch A (2006). Determination of the antimalarial drug artemether in the pharmaceutical preparations by differential pulse polarography. J Pharm Biomed Anal.

